# Supplementary material for: Carafe enables high quality in silico spectral library generation for data-independent acquisition proteomics
Source: bioRxiv. 2024 Oct 18:2024.10.15.618504. Preprint. [Version 1] doi: 10.1101/2024.10.15.618504 (PMC11507862; doi:10.1101/2024.10.15.618504)
Supplement: Supplementary file 1 [file media-2.pdf]

Supplement to "Carafe enables high quality *in silico* spectral library generation for data-independent acquisition proteomics"

Bo Wen[1], Chris Hsu[1], Wen-Feng Zeng[2], Michael Riffle[1], Alexis Chang[1], Miranda Mudge[1], Brook Nunn[1], Matthew D. Berg[1], Judit Villén[1], Michael J. MacCoss[*1], and William S. Noble[*1,3]

[1]Department of Genome Sciences, University of Washington
[2]Department of Proteomics and Signal Transduction, Max Planck Institute of Biochemistry, Germany
[3]Paul G. Allen School of Computer Science and Engineering, University of Washington

[*]Correspondence: maccoss@uw.edu, wnoble@uw.edu

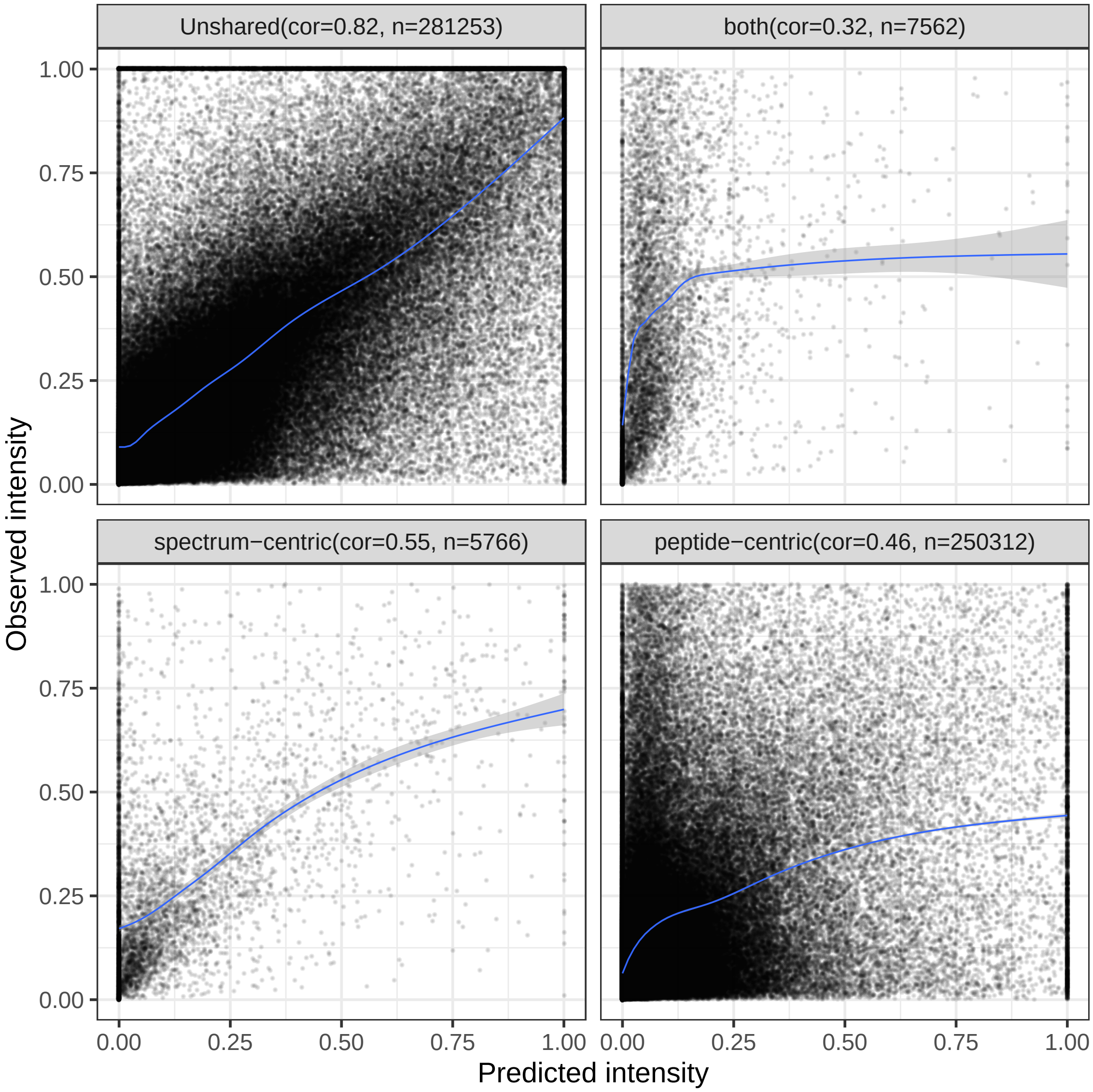

Figure S1: **The performance of the shared peak detection methods.** The detected peaks were divided into four groups: detected as interfered by one method, the other, both or neither (unshared). Each panel plots the predicted versus observed peak intensities, and the blue lines represent a smooth trend calculated using a loess method, highlighting the general pattern of correlation across the intensity range. The number of points and the Pearson correlation between observed and predicted fragment ion intensities is shown at the top of each panel.
